# How the Nature of an Alpha-Nucleophile Determines a Brønsted Type-Plot and Its Reaction Pathways. An Experimental Study

**DOI:** 10.3389/fchem.2021.740161

**Published:** 2022-02-02

**Authors:** Paola R. Campodónico, Ricardo A. Tapia, Cristian Suárez-Rozas

**Affiliations:** ^1^ Centro de Química Médica, Instituto de Ciencias e Innovación en Medicina, Clínica Alemana Universidad del Desarrollo, Santiago, Chile; ^2^ Facultad de Química y de Farmacia, Pontificia Universidad Católica de Chile, Santiago, Chile

**Keywords:** SNAr reactions, mechanisms, hydrogen bond interaction, reactivity, brønsted type-plots

## Abstract

The reactions between 2-chloro-5-nitro pyrimidine with a serie of α-nucleophile derivatives were kinetically evaluated. The kinetic study was carried out in aqueous media and the data shown an unusual split on the Brønsted type-plot, opening a controversial discussion based on reactivities and possible reaction pathways. These split Brønsted type-plots are discussed over the hypothetical transition state (TS) structures associated to concerted or stepwise mechanisms with emphasis on hydrogen bond interactions between electrophile/nucleophile pair able to determine the reactivities and the plausible reaction routes.

## Introduction

The alpha effect accounts for the increased nucleophilic strength due to the presence of an adjacent atom to the nucleophilic center with a lone pair of electrons (Jencks and Carriuolo, 1960a, 1960b; Edwards and Pearson, 1962; Dixon and Bruice, 1972; Buncel and Um, 2004; Kirby et al., 2005; Ren and Yamataka, 2007; 2009; Ormazábal-Toledo et al., 2013b; Kool et al., 2014; Kölmel and Kool, 2017) The nucleophilic strength has been frequently related with the basicity of the nucleophile. However, sometimes the nucleophilicity is greater than the basicity (Anderson and Jencks, 1960) The nucleophilicity concept is associated to electron-rich species (nucleophiles), at the same way, the electrophilicity to electron-deficient species (electrophile) (Ingold, 1929, 1933, 1934) Both concepts are based on electron theory of Lewis (Lewis, 1923) and the general acid-base theory of Brönsted and Lowry (Brönsted, 1923; Lowry, 1923) Then, nucleophilicity/electrophilicity have been used as quantitative scales in order to rationalize the chemical reactivity (Contreras et al., 2003)

The term “α-effect” was used by Edwards and Pearson in order to describe an additional factor relative to the polarizability that influences the nucleophilicity (Edwards and Pearson, 1962) Currently, there are different hypotheses about this effect, such as: 1) increased polarization of the nucleophiles; 2) stabilization of the Transition State (TS) structures along the of the Potential Energy Surface (PES) by the lone pair at α position; 3) relative stability of the reaction products and 4) ground state destabilization due to electron-electron repulsion (Anderson and Jencks, 1960; Edwards and Pearson, 1962; Dixon and Bruice, 1972; Bell et al., 1974; Fountain et al., 2003; Um et al., 2006; Gallardo-Fuentes et al., 2014) Hudson *et al* showed that the magnitude of the α-effect will increase with larger $\beta_{nuc}$ values from Brønsted type-plots (Filippini and Hudson, 1972; Buncel et al., 1993; Fountain et al., 2003) Furthermore, the α-effect is highly modulated by the solvent, but the effect of solvation on the ground state could not explain the changes in the α-effect at higher concentrations of DMSO (Um et al., 1998, 2006) Studies in gas phase have shown that an enhanced α-effect is observed with: 1) high electron density at the α-atom and high electrophilicity values of the electrophile and 2) electronegative α-atom adjacent to the nucleophilic center. However, α-electron withdrawing group diminishes the α-effect (Evanseck et al., 1987; Ren and Yamataka, 2006, 2007; Nigst et al., 2012) Finally, TS structures analysis have shown that there is no difference between nucleophiles with and without α-effect (Ren and Yamataka, 2007)

Therefore, it is possible that the α-effect could be related with several factors, and more studies are needed to provide a detailed description about how this significant effect operates. For better understanding the α-effect, in the present work we studied the magnitude of the α-effect of the reacting pair (electrophile/nucleophile) evaluating the nucleophilic rate coefficients of a nucleophilic aromatic substitution (S_N_Ar) reaction in aqueous media (Cho et al., 2014) The postulated mechanism for a S_N_Ar reaction involves a nucleophilic addition followed by elimination of a leaving group (LG) and it requires the presence of at least one strong electron-withdrawing (Bunnett and Morath, 1955; Liebman et al., 1996) substituent in the ring of the electrophile to stabilize the intermediate, called Meisenheimer Complex (MC) and good LG (Bunnett and Zahler, 1951; Banjoko and Babatunde, 2004; Crampton et al., 2004, 2007; Um et al., 2007; Terrier, 2013; Ormazábal-Toledo et al., 2013b; Gallardo-Fuentes et al., 2014; Gazitúa et al., 2014; Contreras et al., 2015; Calfumán et al., 2017; 2018; Sánchez et al., 2018b) The first step of the reaction mechanism corresponds to the formation of a MC. In a second step, the LG detaches after an intramolecular proton transfer (RLPT) from the nucleophile(Bernasconi and De Rossi, 1976; Ma̧kosza, 1993; Bernasconi et al., 2004; Nudelman, 2009; Um et al., 2012; Ormazabal-Toledo et al., 2013; Ormazábal-Toledo et al., 2013a; Swager and Wang, 2017) Scheme 1 shows the general reaction mechanism for a S_N_Ar reaction. However, more recently, a concerted mechanism has been postulated for this type of reactions. In many cases, the nucleophilic attack on the ipso carbon at the aromatic ring occurs concertedly with the LG departure within a single stepwise pathway without a MC formation (Terrier, 2013; Neumann et al., 2016; Calfumán et al., 2017; Neumann and Ritter, 2017; Stenlid and Brinck, 2017; Kwan et al., 2018; Campodónico et al., 2020) The literature summarizes the mechanistic trends based on the chemical nature of substrates and nucleophiles(Ormazábal-Toledo et al., 2013b; Gazitúa et al., 2014; Alarcón-Espósito et al., 2015, 2016, 2017; 2018; Sánchez et al., 2018a; Campodónico et al., 2020) However, few articles highlight the stabilization of the species along the PES based on hydrogen bond (HB) interactions of the reacting pair (Newington et al., 2007; Ormazábal-Toledo et al., 2013a, 2013b; Gallardo-Fuentes et al., 2014; Calfumán et al., 2017; Sánchez et al., 2018b)

**SCHEME 1:**
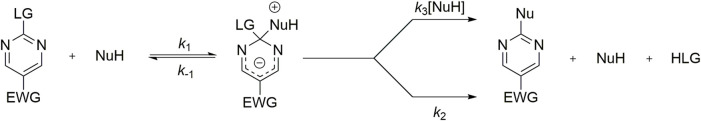
General reaction mechanism for a S_N_Ar with a hypothetical protonated nucleophile. LG corresponds to the Leaving Group and EWG corresponds to electron withdrawing groups.

**SCHEME 2:**
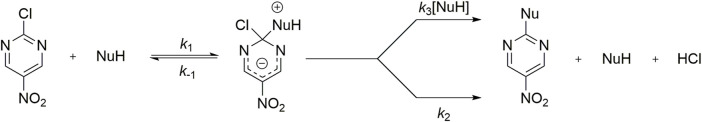
General reaction mechanism for a S_N_Ar between 2-chloro-5-nitro pyrimidine with a hypothetical protonated nucleophile.

In this work, we studied the reaction of 2-chloro-5-nitro pyrimidine (electrophile) with the family of α-nucleophiles depicted in Table 1 (see bottom in Results and Discussion) in aqueous media. Scheme 2 describes the S_N_Ar reaction between 2-chloro-5-nitro pyrimidine and a hypothetical alpha-nucleophile. The main goal was to determine the α-effect on the studied reaction considering the kinetic results and the analysis of the Brønsted type-plot in addition to chemical structures analysis of the reacting pairs.

**TABLE 1:** Summary of nucleophiles and their $pK_{a}$ values in water and $k_{N}$ values for the nucleophile series with 2-chloro-5-nitro pyrimidine.

α-nucleophiles			
Formula	Name	$pK_{a}$	$k_{N}$ (M^−1^s^−1^)
NH_2_NH_2_	Hydrazine	8.10	3.16 ± 0.08
CH_3_NH(OH)	*N*-methylhydroxylamine	6.18	4.66 ± 0.12
NH_2_OH	Hydroxylamine	5.94	0.23 ± 0.001
(CH_3_)_2_NOH	*N,N*-dimethyl Hydroxylamine	5.20	2.06 ± 0.08
CH_3_NH(OCH_3_)	*N,O*-dimethylhydroxylamine	4.75	0.45 ± 0.01
CH_3_ONH_2_	Methoxylamine	4.62	0.02 ± 8.21 × 10^−4^

A Brønsted plot corresponds to a free energy relationship that correlates the logarithm of the nucleophilic rate coefficients ( $k_{N}$ ) and the $pK_{a}$ values of the nucleophiles from Brønsted Equation:

$$logk_{N}=\beta_{nuc}\;pK_{a}+log\;G\;$$ (1)

where G is a constant that depends of the solvent and temperature and $\beta_{nuc}$ corresponds to the development of charge between the reaction sites of the nucleophile/electrophile pair, respectively, along to the PES. (Brönsted and Pedersen, 1924) Therefore, $\beta_{nuc}$ gives information about the TS structure related to the rate determining step (RDS) in the reaction mechanism. (Buncel et al., 1993)

## Materials and Methods

### Reactants

2-Chloro-5-nitro pyrimidine and all the nucleophiles were of the highest quality available commercial products by Sigma Aldrich and Merck. The certificate of analysis guarantees purity ≥99%.

### Kinetic Measurements

The kinetics were carried out spectrophotometrically by means of a diode array spectrophotometer in aqueous media, monitoring the appearance of 2,4-dinitrophenoxide anion at 360 nm. The experimental conditions were 25.0 ± 0.1°C, ionic strength 0.2 M (KCl), at three different pH values maintained by partial protonation of the nucleophiles. All the reactions were studied under excess of the nucleophile at least 10 times greater than the substrate concentration (Um et al., 2007, 2012) in order to achieve pseudo-first-order kinetic conditions. The reactions were started by injection of a substrate stock solution 0.1 M in acetonitrile (10 μl) into the amine solution (2.5 ml in the spectrophotometric cell) reaching a concentration of 0.0004 M in the cell. The formation of colored amino-substituted nitropyrimidine compounds were monitored by UV–vis spectroscopy. In all runs, the pseudo-first-order rate constant ( $k_{obs})$ was found for all the reactions. The $k_{obs}$ were determined by means of the spectrophotometer kinetic software for first order reactions at the wavelength corresponding to the kinetic products. Note that, in aqueous media each pH values correspond to: pH = $pK_{a}$ and 0.3 units up and down in order to analyze the possibility of acid and/or basic catalysis by the reaction media. On the other hand, a Brønsted type-plot requires a broad range of $pK_{a}$ values for the nucleophiles. For this reason, in this study was used a family of nucleophiles with similar chemical features. Then, the relationships between $k_{obs}$ *vs* [Nu] (nucleophile concentration) should be straight lines or straight lines with smooth deviations, which will discard a catalysis processes by the media. See more details in Supplementary Figures S1-S6 and Supplementary Tables S1-S18, respectively in Supplementary Material (SM). This kinetic methodology was taken from previous kinetic studies cited in literature and previous works performed by our group (Castro et al., 1999; Castro et al., 2007; Um et al., 2007; Ormazábal-Toledo et al., 2013, 2013a, 2013b; Gallardo-Fuentes et al., 2014; Gazitúa et al., 2014; Alarcón-Espósito et al., 2015, 2016,2017; Calfuman et al., 2017; 2018; Sánchez et al., 2018a, 2018b; Campodonico et al., 2020)

### Product Analysis

In the studied reactions, the increase of a band centred in the range of 330—550 nm was observed; attributed to the corresponding reaction products for all nucleophile series studied.

### Synthesis of Products

#### 5-Nitro-*N*-phenylpyrimidin-2-amine

To a solution of 2-chloro-5-nitropyrimidine (40 mg, 0.25 mmol) in CH_3_CN (1.0 ml), was added aniline (23.3 mg, 0.25 mmol). The reaction mixture was stirred for 4 h at room temperature, the solvent was removed under vacuum to give a yellow solid which was recrystallized from ethanol (35 mg, 65%), mp 201.5–202.5°C (Lit (Von Bebenburg and Thiele, 1970) 202–203°C). 1H-NMR (400 MHz, DMSO-d6) d: 7.13 (t, J = 7.5 Hz, 1H), 7.36 (t, J = 8.0 Hz, 2H), 7.76 (d, J = 8.0 Hz, 2H), 9.22 (s, 2H), 10.84 (s, 1H); 13C-NMR (100 MHz, DMSO-*d*_6_) d: 126.0, 129.2, 133.9, 140.3, 143.6, 160.3, 166.0.

#### 2-Hydrazinyl-5-Nitropyrimidine

Using the above procedure, from 2-chloro-5-nitropyrimidine (40 mg, 0.25 mmol) and hydrazine (8.0 mg, 0.25 mmol), was obtained a yellow solid (30 mg, 77%), mp 170–172°C (Lit (Caton and McOmie, 1968) 168–169°C). 1H-NMR (400 MHz, DMSO-d6) d: 9.13 (s, 1 H), 9.20 (s, 1 H), 10.84 (s, 1 H); 13C-NMR (100 MHz, DMSO-*d*_6_) d: 136.3, 155.9, 164.3.

## Results and Discussion

In the experimental conditions used only one product formation was spectrophotometrically observed for all the reactions studied. Therefore, the possibility of nucleophilic attack at the unsubstituted ring positions is discarded (Um et al., 2007) This fact was confirmed by synthesis and study of the reaction product (see Experimental Section and SM), discarding the possibility of nucleophilic attack at the unsubstituted positions on the aromatic ring (4 and 6, positions).

The values of $k_{obs}$ for all the reactions are in accordance with Eq. 2 where $k_{0}$ and $k_{N}$ are the rate coefficients for hydrolysis and aminolysis, respectively. Then, the $k_{obs}$ values were obtained at different concentrations of the nucleophile in aqueous media. The $k_{obs}$ values were plotted *vs* [Nu] in order to obtain $k_{N}$ values from Eq. 2:

$$k_{obs}=\;\;k_{0}\;+\;k_{N}\left[Nu\right]$$ (2)

The $k_{obs}\;$ for the reactions can be expressed as Eq. 3(Terrier, 2013; Contreras et al., 2015) and $k_{1}$ , $k_{2}$ and $k_{3}$ are the micro-constants associated to the reaction mechanism of an S_N_Ar reaction (see Scheme 1 and Scheme 2) and obtained applying the steady-state approximation to the S_N_Ar mechanisms:

$$k_{obs}=\frac{\left(k_{1}k_{2}\left[N\right]\;+\;k_{1}k_{3}{\left[Nu\right]}^{2}\right)}{\left(k_{-1}\;+\;k_{2}\;+\;k_{3}\left[Nu\right]\right)}$$ (3)

Linear plots of $k_{obs}\;$ *vs* free nucleophile concentration ( $\;\left[Nu\right]{\;}_{F}$ ) that pass through the origin, suggest that the contribution of the solvent to the $k_{obs}$ values is negligible and the reactions occurs *via* a non-catalyzed route ( $k_{2}$ route in Scheme 1).(Um et al., 2007; Gazitúa et al., 2014; Sánchez et al., 2018b) Thus, $k_{obs}\;$ can be expressed as Eq. 4, where $k_{N}$ is determined from the slope of the linear plots, where $k_{-1}$ + $k_{2}$ >>> $k_{3}$ [Nu] .

$$k_{obs}\;=\;k_{N}\left[Nu\right],\;where\;k_{N}\;=\;\frac{k_{1}k_{2}}{k_{-1}\;+\;k_{2}}$$ (4)

The values of $k_{N}$ and $pK_{a}$ are summarized in Table 1 (kinetic details are in Experimental Section and SM). In order to have a reasonable set of nucleophiles of varying basicity (broad range of $pK_{a}$ values) and nucleophilicity, $pK_{a}$ data were taken from the literature (Kirby et al., 2008) The $k_{N}$ and $pK_{a}$ values from Table 1 were statistically corrected with *p* and *q* parameters, where *q* is the number of equivalent basic sites on the free nucleophile, and *p* is the number of equivalent dissociable protons on the conjugate acid of the nucleophile (Bell, 1973) The values accompanying $k_{N}$ in Table 1 correspond to the error associated to the slope to obtain these kinetic coefficient values.

A preliminary inspection of Table 1 reveals that the general trend in reactivity is: *N-methyl hydroxylamine > hydrazine > N,N-dimethyl hydroxylamine > N,O-dimethyl hydroxylamine > hydroxylamine > methoxylamine.* Note that, this trend is not in agreement with the $pK_{a}$ values of the α-nucleophiles. These α-nucleophiles that have a lone pair vicinal to the attacking nitrogen atom, should display an enhanced nucleophilicity towards 2-chloro-5-nitro pyrimidine. However, the kinetic data showed that the α-effect in this case is not high. This fact suggests that the solvent has a significant effect over the reaction (Buncel and Um, 2004) Note that, water is a molecule with high capacity to establish HB donor/acceptor, then water molecules could be decreasing the nucleophilicity of these α-nucleophiles.

**FIGURE 1:**
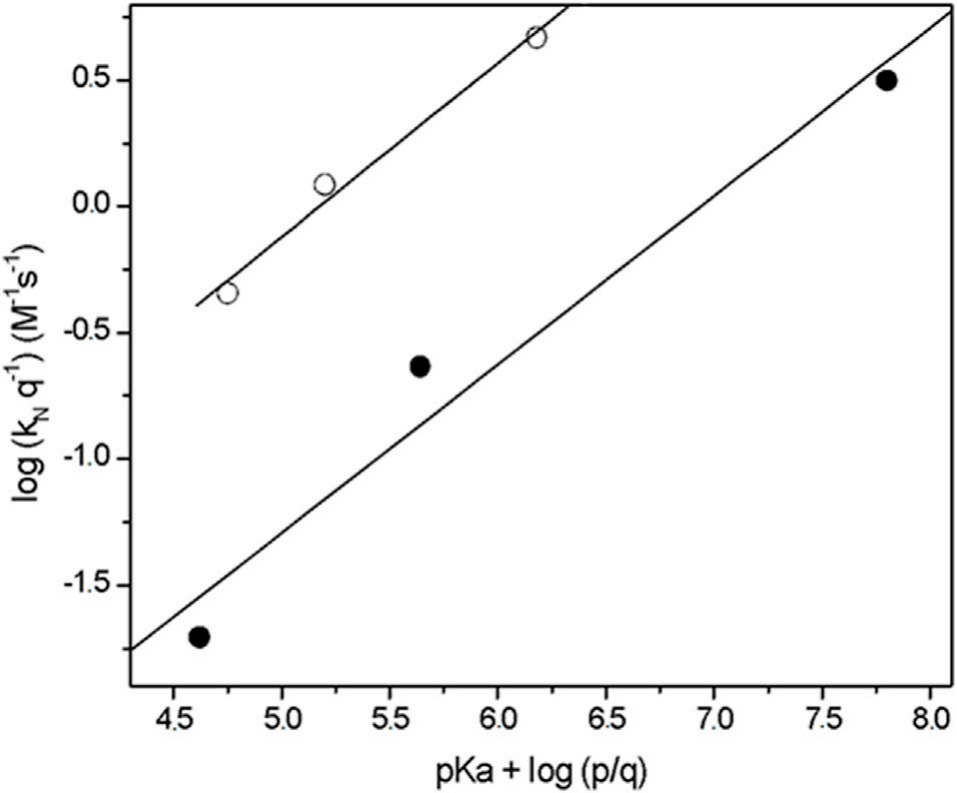
Brønsted -type plots (statistically corrected) obtained for the reactions of 2-choro-5-nitro pyrimidine with alpha nucleophile series in aqueous solution, at 25.0°C and ionic strength of 0.2 M in KCl. In increasing order of $pK_{a}$ : empty circles correspond to *N,O*-dimethyl hydroxylamine, *N*,*N*-dimethyl hydroxylamine and N-methyl hydroxylamine compounds; and full circles correspond to: methoxylamine, hydroxylamine and hydrazine compounds.

Figure 1 shows the statistically corrected Brønsted-type plot for the studied reactions, and the nucleophile serie do not follow the same trend. *Unusually, the Brønsted-type plot is split in two trends, but three points in each one is not enough to establish a correlation and to establish the rate-determining step (RDS* *) of the reaction mechanism.* However, in a first approach a split Brønsted-type plot would suggest that: 1) the studied nucleophile serie have TS structurally different and they should be associated to RDS of the reaction mechanism and 2) the reactivity of the nucleophiles is associated to its chemical structure and steric hindrance close to the nucleophilic center.

**SCHEME 3:**
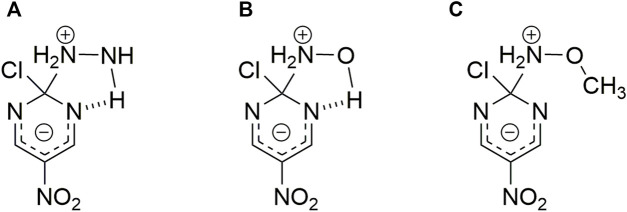
Possible HB interaction between the reacting pair. Structures correspond to hydrazine **(A)**, hydroxylamine **(B)** and methoxylamine **(C)** nucleophiles toward 2-chloro-5-nitro pyrimidine, respectively.(Gallardo-Fuentes et al., 2014)

Then, from Figure 1 is observed an increased order in reactivity for the nucleophiles in both trends in agreement with theirs $pK_{a}$ values. On the other hand, the chemical structure analysis shows the following:

1) The first trend in nucleophilicity is denoted by full circles in Figure 1 that shows the reactivities of *hydrazine > hydroxylamine > methoxylamine* which agrees with their $k_{N}\;$ values. Note that, hydroxylamine is 11.5 times more reactive than methoxylamine and hydrazine 158 times more reactive than methoxylamine. Considering hydrazine as reference compound the influence of the substituent on the nucleophilic reactivity was analyzed. Replacement of one −NH_2_ group in hydrazine by a −OH group reduces the nucleophilicity, and a similar effect is observed replacing one −NH_2_ group in hydrazine by a −MeO group. This trend suggests for hydroxylamine and methoxylamine that the oxygen atom adjacent to the nucleophilic center diminishes the reactivity and that the presence of a −CH_3_ group in methoxylamine diminishes HB ability of the nucleophile. A previous report of the reaction 2-chloro-5-nitro pyrimidine with benzohydrazine derivatives demonstrated that the intramolecular HB enhance the nucleophilicity of these *α-*nucleophiles (Gallardo-Fuentes et al., 2014) Note that, this HB will be formed by hydroxylamine and hydrazine, respectively toward the substrate (see Scheme 3 below). However, methoxylamine does not have the possibility to establish this HB. This specific interaction would be in the TS structure providing information to explain the kinetic behavior of this trend (see Table 1). The synergy of both HB interactions (oriented to electrophilic centre and LG, respectively) would indicate a concerted route. In agreement with the experimental results, the general-base catalyzed mechanism denoted by $k_{3}$ [Nu] in Scheme 1 and Scheme 2 is excluded. Then, the possibility of a stepwise mechanism is still open ( $k_{2}$ channel in Scheme 1 and Scheme 2). *This HB interaction will promote the electron delocalization on the pyrimidine moiety activating the electrophile and nucleophilicity of the α-nucleophile. Then, the nucleophilicity of the α-nucleophile added to the high nucleofugality of the LG of the heterocyclic ring suggests that the MC intermediate is not stable and the reaction mechanism proceeds through one TS structure and a concerted route is suggested*. It is interesting to note that Kwan et al. recently suggested that heterocycles that contain nitrogen atoms and good LG follow a concerted trend (Kwan et al., 2018) Furthermore, Campodonico et al. proposed a concerted mechanism for the reaction of 2-chloro-5-nitro pyrimidine with primary and secondary alicyclic amines (Campodónico et al., 2020) Moreover, Bernasconi et al. postulated that the existence of an intramolecular HB between a hydrogen atom of the nucleophilic centre (amine) and the *o*-NO_2_ group of the substrate could explain the reactivity trend (Bernasconi et al., 2004; Ormazábal-Toledo et al., 2013b) In addition, computational reports based on experimental studies emphasize the role of HB on activating the reacting pair (electrophile/nucleophile) and stabilizing the TS (Bunnett and Morath, 1955; Zingaretti et al., 2003; Bernasconi et al., 2004; Gordillo et al., 2007; Alvaro et al., 2011; Ormazábal-Toledo et al., 2013b; Gallardo-Fuentes et al., 2014; Rohrbach et al., 2020)

2) The second trend (empty circles in Figure 1) shows the following order of reactivity: *N-methyl hydroxylamine > N,N-dimethyl hydroxylamine > N,O-dimethyl hydroxylamine*. This trend shows the decreasing effect of methyl groups on the nucleophilic reactivity; *N*-methylhydroxylamine is 2.3 times more reactive than *N,N*-dimethylhydroxylamine, which in turns is 4.6 times more reactive than *N*,*O*-dimethylhydroxylamine.

**SCHEME 4:**
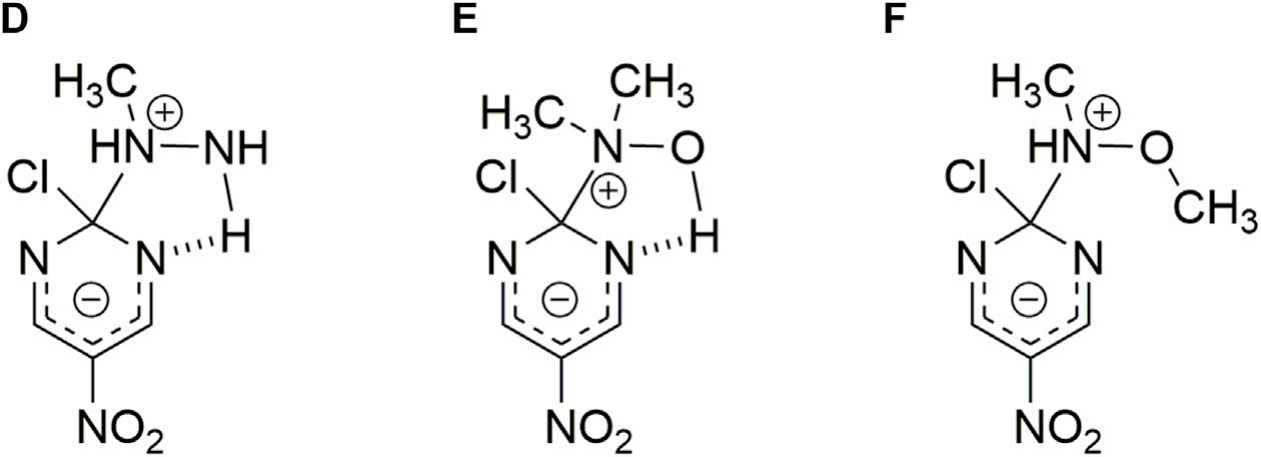
Possible HB interaction between the reacting pair. Structures correspond to *N-*methyl hydroxylamine **(D)**, *N,N-*dimethyl hydroxylamine **(E)** and *N,O*-dimethyl hydroxylamine **(F)** nucleophiles toward 2-chloro-5-nitro pyrimidine, respectively. (In analogy to Scheme 2) (Gallardo-Fuentes et al., 2014)

The comparison between both trends shown an increase in reactivity for the second trend (see Figure 1). For instance, *N,N*-dimethyl hydroxylamine and hydroxylamine have similar $pK_{a}$ values, but the first increased its rate coefficient value in 9 times. *This fact suggests that the inductive effect of methyl group on these structures play a key role in the reactivity of this trend stabilizing the ammonium cation in the TS structures, enhancing the reactivity of the nucleophiles promoting the nucleophilic attack. But, this stabilizing effect could be diminished by steric hindrance in N,N-*dimethyl hydroxylamine. The observed effects that methyl groups increase the nucleophilicity of the substituted nitrogen and decrease the reactivity of the adjacent center was described before by Nigst et al. (Nigst et al., 2012) *Furthermore, the HB interaction, is activating the electrophile and nucleophilicity of the α-nucleophile, except in N,O*-dimethyl hydroxylamine*.* Thus, in this second trend the nucleophilicity strength is higher toward the substrate. See Scheme 4.

In order to reinforce the hypothesis that stereo-electronic effects on TS stabilization*,* may activate the electrophile and to improve the nucleophilicity of the nucleophile, a kinetic study of a serie of anilines using the same substrate was performed. With this purpose, the stereo-electronic effect of electron-donors (-NH_2_, -OMe, -Me) and one electron-acceptor (Me-C=O) groups in the nucleophile, was studied. Table 2 summarize the values of $k_{N}$ and $pK_{a}$ (kinetic details are in Experimental Section and SM). Plots of $k_{obs}$ *vs* [Nu] shown straight lines in accordance with Eq. 3, thereby indicating that the reaction proceeds through a non-catalyzed mechanism (*k*_2_ channel in Scheme 1 and Scheme 2 and Supplementary Figures S7-S12 and Supplementary Tables S19-S26 for more details in SM). The $pK_{a}$ data were taken from the literature in order to have a reasonable set of nucleophiles of varying basicity and nucleophilicity (Castro et al., 1999) The $k_{N}$ and $pK_{a}$ values from Table 2 were statistically corrected with *p* and *q* parameters, respectively (Bell, 1973) The values accompanying $k_{N}$ in Table 2 correspond to the error associated to the slope, respectively to obtain these kinetic coefficient values.

**TABLE 2:** Aniline serie and their $pK_{a}$ values in water and $k_{N}$ values for the nucleophile series with 2-chloro-5-nitro pyrimidine.

Nucleophiles	$pK_{a}$	*k*_*N*_ (M^−1^s^−1^)
4-phenylenediamine	6.20	33.7 ± 0.0610
4-methoxyaniline	5.65	7.33 ± 0.140
4-methylaniline	5.08	2.49 ± 0.00611
Aniline	4.73	0.99 ± 0.0139
3-methoxyaniline	4.36	0.627 ± 0.0133
3-aminoacetophenone	3.64	0.266 ± 0.000954

**FIGURE 2:**
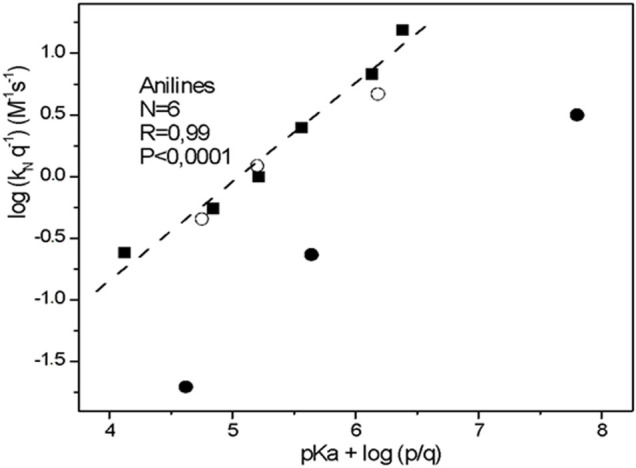
Brønsted -type plot (statistically corrected) obtained for the reactions of 2-choro-5-nitro pyrimidine with aniline series in aqueous solution, at 25.0°C and ionic strength of 0.2 M in KCl (full square). The empty circles correspond to: *N*,*O*-dimethyl hydroxylamine, *N*,*N*-dimethyl hydroxylamine and N-methyl hydroxylamine compounds and full circles correspond to: methoxylamine, hydroxylamine and hydrazine compounds, respectively (see Figure 1).

Figure 2 shows a Brønsted type-plot with a $\beta_{nuc}$ value of 0.83 for the aniline serie (full squares). This value suggests that the bond formation between the nucleophile (aniline derivatives) and the substrate is fully advanced in the rate-limiting TS. This value agrees with the $\beta_{nuc}$ value reported for the S_N_Ar reaction between 2,4-dinitrophenylsulfonylchloride with secondary alicyclic (SA) amines in aqueous media, where the LG departure was attributed as the RDS for a non-catalyzed pathway (Gazitúa et al., 2014) *This fact, would suggest a stepwise route where the LG departure is the RDS on the reaction mechanism for the aniline serie. Then, the unusual split* Brønsted*-type plot for the alpha nucleophile above (second trend for empty circles in* Figure 1 *) reinforce the idea that it will be associated to a change on the reaction pathway for the studied nucleophile series toward the substrate; suggesting a stepwise mechanism were the RDS is LG departure (See* Figure 2 *).*

**SCHEME 5:**
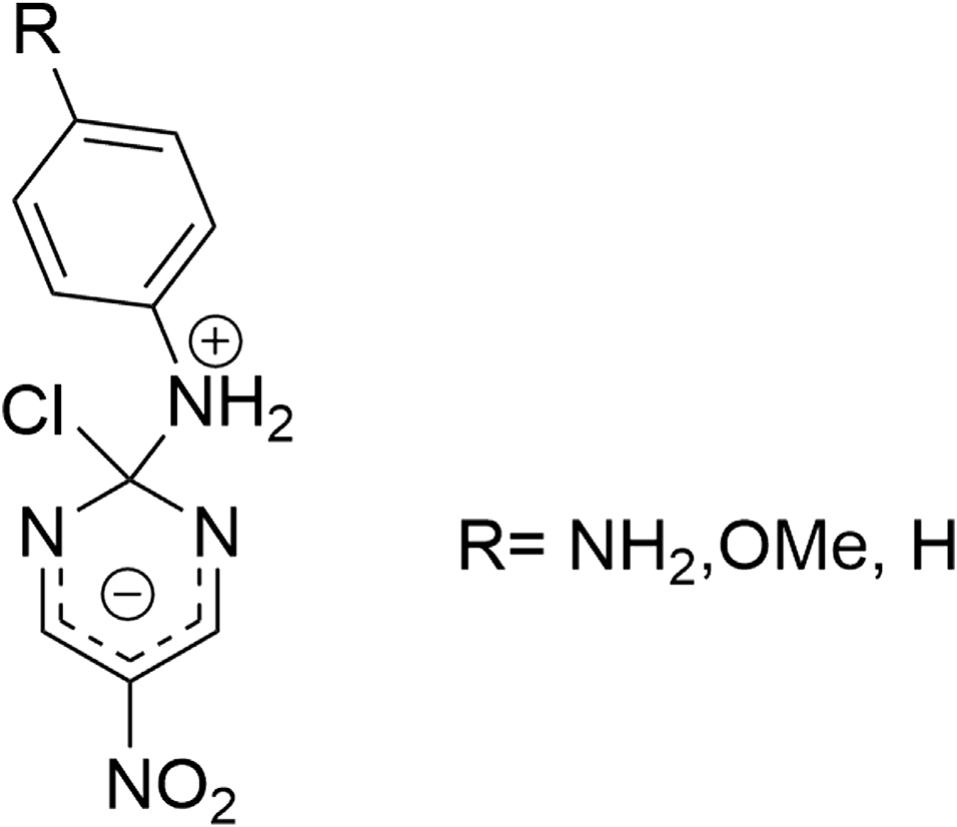
A general scheme of anilinium cation TS structure.

Focusing our analyses over the chemical structure of the aniline serie; the rate coefficients are notably sensitive to the inductive effects of the substituents. Thus, electron-donating *p*-substituent has a strong effect on the nucleophilicity and the reactivity order for the nucleophiles agrees with theirs $pK_{a}$ values (see Table 2). For instance, 4*-*phenylenediamine ( $pK_{a}\;$ = 6.20) has the highest nucleophilic rate coefficient and 3-aminoacetophenone ( $pK_{a\;}$ = 3.64) the lowest. Therefore, electron-donating substituent plays an important role on the stabilization of the positive charge on the anilinium cation TS structure (see Scheme 5 above). Then, hydrogen-bonding interactions of the media (solvent as acceptor with *β* parameter) with positive charge on the activated complex of the reaction will stabilize the activated complex better than the reactants; therefore, increasing the *β* parameter accelerates the reaction rate (Kamlet et al., 1983)

Accordingly, heterocyclic substrates that contains nitrogen atoms in its chemical structure assist a favorable nucleophilic attack by high nucleophilic amines, but slow LG departures. On this way, the nature of the reacting pair and the reaction media drastically affects the nucleophilic reaction rates and the RDS on the reaction mechanism (Klopman and Frierson, 1984; Garver et al., 2011; Gazitúa et al., 2018; Um et al., 2018)

Finally, in order to determine the HB effect, it was carried out the kinetic study of phenyl hydrazine (see Supplementary Tables S37-S39 and Supplementary Figure S13 in SM) with the same substrate. Note that, this nucleophile with a potential alpha-effect showed a similar behaviour than aniline derivatives ( $k_{N}$ = 1.45 ± 0.006 M^−1^s^−1^ and $pK_{a}$ = 5.25 *versus* $k_{N}$ = 0.99 ± 0.0139 M^−1^s^−1^ and $pK_{a}$ = 4.73, respectively) reinforcing the substituent effect exerted over the nucleophilic center. Moreover, is interesting to analyze the nucleophilic rate values for phenyl hydrazine and *p-*phenylenediamine compounds ( $k_{N}$ = 1.45 ± 0.006 M^−1^s^−1^ and $pK_{a}$ = 5.25 versus $k_{N}$ = 33.7 ± 0.0610 and $pK_{a}$ = 5.25, respectively) (Brighente and Yunes, 1997) Therefore, in the aniline serie, the fundamental role is played by the inductive effect of the substituents increasing the nucleophilicity and stabilizing the anilinium cation TS structure.

## Concluding Remarks

A complete experimental study on an S_N_Ar reaction has been presented. The experimental results shown an unusual broken on the Brönsted type-plot for the alpha nucleophiles studied, suggesting TS structures structurally different given by the reactivities associated to the chemical structure of them: First, an HB interaction is suggested between the α-hydrogen atom of the nucleophile which is oriented toward the nitrogen atom of the pyrimidine moiety. This HB will promote the reactivity of this serie. Then, a second HB oriented towards the LG, added to the chemical features of the reacting pairs, suggest a concerted route. The second family of alpha-nucleophiles showed a key role of the methyl group inductive effect, stabilizing the ammonium cation in the TS structures, and increasing the reactivity of the nucleophiles. Then, a complete kinetic study based on aniline derivatives toward the same electrophile in order to analyze the Brönsted type-plot, observing a high $\beta_{nuc}$ value. This value suggests that the bond formation between the aniline derivatives and the substrate is fully advanced in the rate-limiting TS and LG departure is the RDS for a non-catalyzed pathway. On the other hand, the stereo-electronic effects on TS stabilization shows that an electron-donating substituent plays an important role on the stabilization of the positive charge on the anilinium cation TS structure accelerating the nucleophilic attack. In summary, the magnitude of the alpha effect depends on the chemical structure of the nucleophiles added to solvent effect, and particularly the possibility to stablish HB interactions between the reacting pair. Then, a detailed experimental study must consider all the factors that are contributing to the reactivity and determining the reaction pathway. An interesting point will be to test these reactions in an aprotic solvents and/or non-conventional solvent such as deep eutectic solvent or an ionic liquid.

## Data Availability

The original contributions presented in the study are included in the article/Supplementary Material, further inquiries can be directed to the corresponding author.
