# Supplementary material for: How the Nature of an Alpha-Nucleophile Determines a Brønsted Type-Plot and Its Reaction Pathways. An Experimental Study
Source: Front Chem. 2022 Feb 2;9:740161. doi: 10.3389/fchem.2021.740161 (PMC8847609; doi:10.3389/fchem.2021.740161)

Supplementary Material

How the nature of an alpha-nucleophile determines a Brönsted type-plot and its reaction pathways.

Paola R. Campodónico*[1] , Ricardo A. Tapia[2] and Cristian Suárez-Rozas[1]

[1]Centro de Química Médica. Facultad de Medicina. Clínica Alemana Universidad del Desarrollo. Código Postal 7710162. Santiago, Chile. [2]Facultad de Química y de Farmacia, Pontificia Universidad Católica de Chile, Código Postal 6094411, Santiago, Chile.

pcampodonico@udd.cl

Supplementary Figures and Tables

Table S1	Kinetic data for the reaction of pyrimidine with hydrazine in aqueous solution at 25°C±0.1°C and pH=7.81.	S6
Table S2	Kinetic data for the reaction of pyrimidine with hydrazine in aqueous solution at 25°C±0.1°C and pH=8.11.	S6
Table S3	Kinetic data for the reaction of pyrimidine with hydrazine in aqueous solution at 25°C±0.1°C and pH=8.41.	S6
Table S4	Kinetic data for the reaction of pyrimidine with N-methyl hydroxylamine in aqueous solution at 25°C±0.1°C and pH=5.9.	S7
Table S5	Kinetic data for the reaction of pyrimidine with N-methyl hydroxylamine in aqueous solution at 25°C±0.1°C and pH=6.2.	S7
Table S6	Kinetic data for the reaction of pyrimidine with N-methyl hydroxylamine in aqueous solution at 25°C±0.1°C and pH=6.5.	S8
Table S7	Kinetic data for the reaction of pyrimidine with hydroxylamine in aqueous solution at 25°C±0.1°C and pH=5.7.	S8
Table S8	Kinetic data for the reaction of pyrimidine with hydroxylamine in aqueous solution at 25°C±0.1°C and pH=5.96.	S9
Table S9	Kinetic data for the reaction of pyrimidine with hydroxylamine in aqueous solution at 25°C±0.1°C and pH=6.3.	S9
Table S10	Kinetic data for the reaction of pyrimidine with N,N-dimethyl hydroxylamine in aqueous solution at 25°C±0.1°C and pH=4.9.	S10
Table S11	Kinetic data for the reaction of pyrimidine with N,N-dimethyl hydroxylamine in aqueous solution at 25°C±0.1°C and pH=5.2.	S10
Table S12	Kinetic data for the reaction of pyrimidine with N,N-dimethyl hydroxylamine in aqueous solution at 25°C±0.1°C and pH=5.5.	S10
Table S13	Kinetic data for the reaction of pyrimidine with N,O-dimethyl hydroxylamine in aqueous solution at 25°C±0.1°C and pH=4.4.	S11
Table S14	Kinetic data for the reaction of pyrimidine with N,O-dimethyl hydroxylamine in aqueous solution at 25°C±0.1°C and pH=4.7.	S11
Table S15	Kinetic data for the reaction of pyrimidine with N,O-dimethyl hydroxylamine in aqueous solution at 25°C±0.1°C and pH=5.0.	S12

Table S16	Kinetic data for the reaction of pyrimidine with methoxylamine in aqueous solution at 25°C±0.1°C and pH=4.3.	S12
Table S17	Kinetic data for the reaction of pyrimidine with methoxylamine in aqueous solution at 25°C±0.1°C and pH=4.62.	S13
Table S18	Kinetic data for the reaction of pyrimidine with methoxylamine in aqueous solution at 25°C±0.1°C and pH=4.9.	S13
Table S19	Kinetic data for the reaction of pyrimidine with 4-phenylendiamine in aqueous solution at 25°C±0.1°C and pH=5.9.	S14
Table S20	Kinetic data for the reaction of pyrimidine with 4-phenylendiamine in aqueous solution at 25°C±0.1°C and pH=6.2.	S14
Table S21	Kinetic data for the reaction of pyrimidine with 4-phenylendiamine in aqueous solution at 25°C±0.1°C and pH=6.5.	S15
Table S22	Kinetic data for the reaction of pyrimidine with 4-methoxyaniline in aqueous solution at 25°C±0.1°C and pH=5.35.	S15
Table S23	Kinetic data for the reaction of pyrimidine with 4-methoxyaniline in aqueous solution at 25°C±0.1°C and pH=5.65.	S16
Table S24	Kinetic data for the reaction of pyrimidine with 4-methoxyaniline in aqueous solution at 25°C±0.1°C and pH=5.95.	S16
Table S25	Kinetic data for the reaction of pyrimidine with 4-methylaniline in aqueous solution at 25°C±0.1°C and pH=4.78.	S17
Table S26	Kinetic data for the reaction of pyrimidine with 4-methylaniline in aqueous solution at 25°C±0.1°C and pH=5.08.	S17
Table S27	Kinetic data for the reaction of pyrimidine with 4-methylaniline in aqueous solution at 25°C±0.1°C and pH=5.38.	S18
Table S28	Kinetic data for the reaction of pyrimidine with aniline in aqueous solution at 25°C±0.1°C and pH=4.43.	S18
Table S29	Kinetic data for the reaction of pyrimidine with aniline in aqueous solution at 25°C±0.1°C and pH=4.73.	S19
Table S30	Kinetic data for the reaction of pyrimidine with aniline in aqueous solution at 25°C±0.1°C and pH=5.03.	S19
Table S31	Kinetic data for the reaction of pyrimidine with 3-methoxyaniline in aqueous solution at 25°C±0.1°C and pH=4.06.	S20

Table S32	Kinetic data for the reaction of pyrimidine with 3-methoxyaniline in aqueous solution at 25°C±0.1°C and pH=4.36.	S20
Table S33	Kinetic data for the reaction of pyrimidine with 3-methoxyaniline in aqueous solution at 25°C±0.1°C and pH=4.66.	S21
Table S34	Kinetic data for the reaction of pyrimidine with 3-aminoacetophenone in aqueous solution at 25°C±0.1°C and pH=3.34.	S21
Table S35	Kinetic data for the reaction of pyrimidine with 3-aminoacetophenone in aqueous solution at 25°C±0.1°C and pH=3.64.	S22
Table S36	Kinetic data for the reaction of pyrimidine with 3-aminoacetophenone in aqueous solution at 25°C±0.1°C and pH=3.94.	S22
Table S37	Kinetic data for the reaction of pyrimidine with phenylhydrazine in aqueous solution at 25°C±0.1°C and pH=4.95	S22
Table S38	Kinetic data for the reaction of pyrimidine with phenylhydrazine in aqueous solution at 25°C±0.1°C and pH=5.25	S23
Table S39	Kinetic data for the reaction of pyrimidine with phenylhydrazine in aqueous solution at 25°C±0.1°C and pH=5.55	S23
Figure S1	Plot of k_{obs} against free amine concentration $[RNH_2]_F$ for the reaction of pyrimidine with hydrazine at three pH values in aqueous solution at 25.0 °C and ionic strength 0.2M (KCl).	S24
Figure S2	Plot of k_{obs} against free amine concentration $[RNH_2]_F$ for the reaction of pyrimidine with N-methyl hydroxylamine at three pH values in aqueous solution at 25.0 °C and ionic strength 0.2M (KCl).	S24
Figure S3	Plot of k_{obs} against free amine concentration $[RNH_2]_F$ for the reaction of pyrimidine with hydroxylamine at three pH values in aqueous solution at 25.0 °C and ionic strength 0.2M (KCl).	S24
Figure S4	Plot of k_{obs} against free amine concentration $[RNH_2]_F$ for the reaction of pyrimidine with N,N-dimethyl hydroxylamine at three pH values in aqueous solution at 25.0 °C and ionic strength 0.2M (KCl).	S25
Figure S5	Plot of k_{obs} against free amine concentration $[RNH_2]_F$ for the reaction of pyrimidine with N,O-dimethyl hydroxylamine at three	S25

	pH values in aqueous solution at 25.0 °C and ionic strength 0.2M (KCl).	
Figure S6	Plot of k_{obs} against free amine concentration $[RNH_2]_F$ for the reaction of pyrimidine with methoxylamine at three pH values in aqueous solution at 25.0 °C and ionic strength 0.2M (KCl).	S25
Figure S7	Plot of k_{obs} against free amine concentration $[RNH_2]_F$ for the reaction of pyrimidine with 4-phenylendiamine at three pH values in aqueous solution at 25.0 °C and ionic strength 0.2M (KCl).	S26
Figure S8	Plot of k_{obs} against free amine concentration $[RNH_2]_F$ for the reaction of pyrimidine with 4-methoxyaniline at three pH values in aqueous solution at 25.0 °C and ionic strength 0.2M (KCl).	S26
Figure S9	Plot of k_{obs} against free amine concentration $[RNH_2]_F$ for the reaction of pyrimidine with 4-methylaniline at three pH values in aqueous solution at 25.0 °C and ionic strength 0.2M (KCl).	S26
Figure S10	Plot of k_{obs} against free amine concentration $[RNH_2]_F$ for the reaction of pyrimidine with aniline at three pH values in aqueous solution at 25.0 °C and ionic strength 0.2M (KCl).	S27
Figure S11	Plot of k_{obs} against free amine concentration $[RNH_2]_F$ for the reaction of pyrimidine with 3-methoxyaniline at three pH values in aqueous solution at 25.0 °C and ionic strength 0.2M (KCl).	S27
Figure S12	Plot of k_{obs} against free amine concentration $[RNH_2]_F$ for the reaction of pyrimidine with 3-aminoacetophenone at three pH values in aqueous solution. at 25.0 °C and ionic strength 0.2M (KCl).	S27
Figure S13	Plot of k_{obs} against free amine concentration $[RNH_2]_F$ for the reaction of pyrimidine with phenylhydrazine at three pH values in aqueous solution at 25.0 °C and ionic strength 0.2M (KCl).	S28

Table S1. Kinetic data for the reaction of pyrimidine with hydrazine in aqueous solution at 25°C±0.1°C and pH=7.81.

	10^3 [RNH_2]$_F$	10^2 k_{obs} / s^{-1}
1	2.83	0.724
2	7.07	1.88
3	11.3	3.13
4	15.5	3.80
5	19.8	5.86
6	24.0	7.77

Table S2. Kinetic data for the reaction of pyrimidine with hydrazine in aqueous solution at 25°C±0.1°C and pH=8.11.

	10^3 [RNH_2]$_F$	10^2 k_{obs} / s^{-1}
1	3.96	1.02
2	9.90	2.24
3	15.8	4.03
4	21.8	5.23
5	27.7	6.65
6	33.7	8.60

Table S3. Kinetic data for the reaction of pyrimidine with hydrazine in aqueous solution at 25°C±0.1°C and pH=8.41.

	10^3 [RNH_2]$_F$	10^2 k_{obs} / s^{-1}
1	4.73	1.36
2	11.8	3.14
3	18.9	5.27
4	26.0	7.56
5	33.1	9.34
6	40.2	11.9
7	47.3	13.9

Table S4. Kinetic data for the reaction of pyrimidine with N-methyl hydroxylamine in aqueous solution at 25°C±0.1°C and pH=5.9.

	10^3 $[RNH_2]_F$	10^2 k_{obs} / s^{-1}
1	2.75	1.44
2	6.18	3.12
3	11.0	5.04
4	15.1	8.01
5	19.2	10.0
6	23.4	12.0
7	27.5	14.0

Table S5. Kinetic data for the reaction of pyrimidine with N-methyl hydroxylamine in aqueous solution at 25°C±0.1°C and pH=6.2.

	10^3 $[RNH_2]_F$	10^2 k_{obs} / s^{-1}
1	3.71	1.86
2	8.36	3.81
3	14.9	6.83
4	20.4	9.06
5	26.0	12.2
6	31.6	14.8
7	37.1	17.6

Table S6. Kinetic data for the reaction of pyrimidine with N-methyl hydroxylamine in aqueous solution at 25°C±0.1°C and pH=6.5.

	10^3 $[RNH_2]_F$	10^2 k_{obs} / s^{-1}
1	3.08	1.46
2	6.93	3.49
3	12.3	5.53
4	16.9	7.80
5	21.5	10.0
6	26.2	11.9
7	30.8	13.4

Table S7. Kinetic data for the reaction of pyrimidine with hydroxylamine in aqueous solution at 25°C±0.1°C and pH=5.7.

	10^3 $[RNH_2]_F$	10^3k_{obs} / s^{-1}
1	5.21	1.58
2	13.0	3.38
3	20.8	5.00
4	28.7	6.42
5	36.5	10.1
6	44.3	11.9
7	52.1	14.8

Table S8. Kinetic data for the reaction of pyrimidine with hydroxylamine in aqueous solution at 25°C±0.1°C and pH=5.96.

	10^3 $[RNH_2]_F$	10^3 k_{obs} / s^{-1}
1	6.50	1.52
2	16.2	4.13
3	26.0	6.43
4	35.7	7.65
5	45.5	9.34
6	55.2	11.8
7	65.0	13.6

Table S9. Kinetic data for the reaction of pyrimidine with hydroxylamine in aqueous solution at 25°C±0.1°C and pH=6.30.

	10^3 $[RNH_2]_F$	10^3 k_{obs} / s^{-1}
1	9.31	2.79
2	23.3	5.56
3	37.2	9.51
4	51.2	14.1
5	65.2	16.1
6	79.1	18.7

Table S10. Kinetic data for the reaction of pyrimidine with N,N-dimethyl hydroxylamine in aqueous solution at 25°C±0.1°C and pH=4.9.

	10^3 $[RNH_2]_F$	10^3 k_{obs} / s^{-1}
1	0.63	3.06
2	1.57	5.07
3	2.52	7.98
4	3.46	9.09
5	4.40	10.9
6	5.35	13.5
7	6.29	15.2

Table S11. Kinetic data for the reaction of pyrimidine with N,N-dimethyl hydroxylamine in aqueous solution at 25°C±0.1°C and pH=5.2.

	10^3 $[RNH_2]_F$	10^3 k_{obs} / s^{-1}
1	0.92	3.92
2	2.07	7.42
3	3.67	9.07
4	5.05	11.0
5	6.43	13.8
6	7.80	18.6

Table S12. Kinetic data for the reaction of pyrimidine with N,N-dimethyl hydroxylamine in aqueous solution at 25°C±0.1°C and pH=5.5.

	10^3 $[RNH_2]_F$	10^3 k_{obs} / s^{-1}
1	0.75	3.26
2	1.87	6.41
3	3.00	8.07
4	4.12	11.4
5	5.24	14.4
6	6.37	15.6

Table S13. Kinetic data for the reaction of pyrimidine with N,O-dimethyl hydroxylamine in aqueous solution at 25°C±0.1°C and pH=4.4.

	10^3 $[RNH_2]_F$	10^3 k_{obs} / s^{-1}
1	1.02	0.50
2	2.54	1.31
3	4.07	2.16
4	5.60	2.89
5	7.12	3.62
6	8.65	4.31
7	10.2	5.23

Table S14. Kinetic data for the reaction of pyrimidine with N,O-dimethyl hydroxylamine in aqueous solution at 25°C±0.1°C and pH=4.7.

	10^3 $[RNH_2]_F$	10^3 k_{obs} / s^{-1}
1	1.62	0.70
2	3.64	1.52
3	6.47	2.52
4	8.89	3.93
5	11.3	5.16
6	13.7	5.86
7	16.2	7.12

Table S15. Kinetic data for the reaction of pyrimidine with N,O-dimethyl hydroxylamine in aqueous solution at 25°C±0.1°C and pH=5.0.

	10^3 [RNH_2]$_F$	10^3 k_{obs} / s^{-1}
1	1.69	0.75
2	4.22	1.87
3	6.76	2.94
4	9.29	4.14
5	11.8	5.31
6	14.4	6.32
7	16.9	7.84

Table S16. Kinetic data for the reaction of pyrimidine with methoxylamine in aqueous solution at 25°C±0.1°C and pH=4.3.

	10^3 [RNH_2]$_F$	10^4 k_{obs} / s^{-1}
1	1.58	0.94
2	3.95	1.14
3	6.31	1.66
4	8.68	2.62
5	11.0	3.05
6	13.4	3.46
7	15.8	4.15

Table S17. Kinetic data for the reaction of pyrimidine with methoxylamine in aqueous solution at 25°C±0.1°C and pH=4.62.

	10^3 [RNH$_2$]$_F$	10^4 k_{obs} / s^{-1}
1	2.31	0.71
2	5.19	1.38
3	9.23	2.42
4	12.7	3.17
5	16.2	3.90
6	19.6	---
7	23.07	5.51

Table S18. Kinetic data for the reaction of pyrimidine with methoxylamine in aqueous solution at 25°C±0.1°C and pH=4.90.

	10^3 [RNH$_2$]$_F$	10^4 k_{obs} / s^{-1}
1	3.33	0.76
2	8.33	1.72
3	13.3	2.87
4	18.3	3.86
5	23.3	4.87
6	28.3	5.93
7	33.3	6.68

Table S19. Kinetic data for the reaction of pyrimidine with 4-phenylenediamine in aqueous solution at 25°C±0.1°C and pH=5.9.

	10^3 $[RNH_2]_F$	10^2 k_{obs} / s^{-1}
1	0.214	0.752
2	0.536	1.92
3	0.857	3.21
4	1.18	4.34
5	1.50	5.35
6	1.82	6.64
7	2.14	7.58

Table S20. Kinetic data for the reaction of pyrimidine with 4-phenylenediamine in aqueous solution at 25°C±0.1°C and pH=6.2.

	10^3 $[RNH_2]_F$	10^2 k_{obs} / s^{-1}
1	0.318	0.959
2	0.795	2.41
3	1.27	3.94
4	1.75	5.72
5	2.23	7.63
6	2.70	9.05
7	3.18	10.6

Table S21. Kinetic data for the reaction of pyrimidine with 4-phenylenediamine in aqueous solution at 25°C±0.1°C and pH=6.5.

	10^3 $[RNH_2]_F$	10^2 k_{obs} / s^{-1}
1	0.348	1.04
2	0.870	2.19
3	1.39	3.99
4	1.92	5.53
5	2.44	7.47
6	2.96	8.86
7	3.48	10.7

Table S22. Kinetic data for the reaction of pyrimidine with 4-methoxyaniline in aqueous solution at 25°C±0.1°C and pH=5.35.

	10^3 $[RNH_2]_F$	10^2 k_{obs} / s^{-1}
1	0.235	0.181
2	0.587	0.407
3	0.939	0.709
4	1.29	1.08
5	1.64	1.41
6	2.00	1.73
7	2.35	2.00

Table S23. Kinetic data for the reaction of pyrimidine with 4-methoxyaniline in aqueous solution at 25°C±0.1°C and pH=5.65.

	10^3 $[RNH_2]_F$	10^2 k_{obs} / s^{-1}
1	0.278	0.179
2	0.572	0.381
3	0.817	0.537
4	2.04	1.21
5	3.27	2.19
6	4.49	3.06
7	5.72	3.85

Table S24. Kinetic data for the reaction of pyrimidine with 4-methoxyaniline in aqueous solution at 25°C±0.1°C and pH=5.95.

	10^3 $[RNH_2]_F$	10^2 k_{obs} / s^{-1}
1	0.467	0.287
2	1.17	0.677
3	1.87	1.17
4	2.57	1.62
5	3.27	2.07
6	3.97	2.54

Table S25. Kinetic data for the reaction of pyrimidine with 4-methylaniline in aqueous solution at 25°C±0.1°C and pH=4.78.

	10^3 $[RNH_2]_F$	10^2 k_{obs} / s^{-1}
1	0.189	0.0557
2	0.473	0.128
3	0.756	0.208
4	1.04	0.281
5	1.32	0.389
6	1.61	0.456
7	1.89	0.554

Table S26. Kinetic data for the reaction of pyrimidine with 4-methylaniline in aqueous solution at 25°C±0.1°C and pH=5.08.

	10^3 $[RNH_2]_F$	10^2 k_{obs} / s^{-1}
1	0.261	0.0873
2	0.653	0.204
3	1.05	0.314
4	1.44	0.428
5	1.83	0.465
6	2.22	0.574
7	2.61	0.664

Table S27. Kinetic data for the reaction of pyrimidine with 4-methylaniline in aqueous solution at 25°C±0.1°C and pH=5.38.

	10^3 [RNH_2]$_F$	10^2 k_{obs} / s^{-1}
1	0.350	0.108
2	0.875	0.262
3	1.40	0.402
4	1.93	0.563
5	2.45	0.627
6	2.98	0.763
7	3.50	0.882

Table S28. Kinetic data for the reaction of pyrimidine with aniline in aqueous solution at 25°C±0.1°C and pH=4.43.

	10^3 [RNH_2]$_F$	10^2 k_{obs} / s^{-1}
1	1.72	0.210
2	4.31	0.476
3	6.89	0.819
4	9.48	1.06
5	12.1	1.27
6	14.6	1.51
7	17.2	1.80

Table S29. Kinetic data for the reaction of pyrimidine with aniline in aqueous solution at 25°C±0.1°C and pH=4.73.

	10^3 $[RNH_2]_F$	10^2 k_{obs} / s^{-1}
1	2.52	0.254
2	6.31	0.596
3	10.1	1.00
4	13.9	1.40
5	17.7	1.70
6	21.5	2.10
7	25.2	2.49

Table S30. Kinetic data for the reaction of pyrimidine with aniline in aqueous solution at 25°C±0.1°C and pH=5.03.

	10^3 $[RNH_2]_F$	10^2 k_{obs} / s^{-1}
1	3.41	0.338
2	8.54	0.849
3	13.7	1.42
4	18.8	1.89
5	23.9	2.40
6	29.0	2.91
7	34.1	3.53

Table S31. Kinetic data for the reaction of pyrimidine with 3-methoxyaniline in aqueous solution at 25°C±0.1°C and pH=4.06.

	10^3 $[RNH_2]_F$	10^2 k_{obs} / s^{-1}
1	0.603	0.0405
2	1.51	0.0953
3	2.41	0.175
4	3.32	0.221
5	4.22	0.300
6	5.13	0.365
7	6.03	0.446

Table S32. Kinetic data for the reaction of pyrimidine with 3-methoxyaniline in aqueous solution at 25°C±0.1°C and pH=4.36.

	10^3 $[RNH_2]_F$	10^2 k_{obs} / s^{-1}
1	0.654	0.0606
2	2.33	0.139
3	3.74	0.223
4	5.14	0.312
5	6.54	0.389
6	7.94	0.485
7	9.34	0.568

Table S33. Kinetic data for the reaction of pyrimidine with 3-methoxyaniline in aqueous solution at 25°C±0.1°C and pH=4.66.

	10^3 $[RNH_2]_F$	10^2 k_{obs} / s^{-1}
1	1.25	0.0736
2	3.13	0.167
3	5.01	0.280
4	6.88	0.377
5	8.76	0.466
6	10.6	0.591
7	12.5	0.691

Table S34. Kinetic data for the reaction of pyrimidine with 3-aminoacetophenone in aqueous solution at 25°C±0.1°C and pH=3.34.

	10^3 $[RNH_2]_F$	10^2 k_{obs} / s^{-1}
1	1.03	0.033
2	2.57	0.085
3	4.11	0.117
4	5.66	0.174
5	7.20	0.224
6	8.74	0.241
7	10.3	0.279

Table S35. Kinetic data for the reaction of pyrimidine with 3-aminoacetophenone in aqueous solution at 25°C±0.1°C and pH=3.64.

	10^3 $[RNH_2]_F$	10^2 k_{obs} / s^{-1}
1	1.62	0.046
2	4.05	0.112
3	6.48	0.174
4	8.91	0.241
5	11.3	0.340
6	13.8	0.396
7	16.2	0.454

Table S36. Kinetic data for the reaction of pyrimidine with 3-aminoacetophenone in aqueous solution at 25°C±0.1°C and pH=3.94.

	10^3 $[RNH_2]_F$	10^2 k_{obs} / s^{-1}
1	2.09	0.059
2	5.22	0.151
3	8.35	0.199
4	11.5	0.281
5	14.6	0.365

Table S37. Kinetic data for the reaction of pyrimidine with phenylhydrazine in aqueous solution at 25°C±0.1°C and pH=4.95.

	10^3 $[RNH_2]_F$	10^2 k_{obs} / s^{-1}
1	1.54	0.093
2	3.85	0.279
3	6.16	0.702
4	8.47	1.061
5	10.8	1.520

Table S38. Kinetic data for the reaction of pyrimidine with phenylhydrazine in aqueous solution at 25°C±0.1°C and pH=5.25.

	10^3 [RNH_2]$_F$	10^2 k_{obs} / s^{-1}
1	2.09	0.29
2	5.22	0.87
3	8.34	1.48
4	11.5	2.02
5	14.6	2.50

Table S39. Kinetic data for the reaction of pyrimidine with phenylhydrazine in aqueous solution at 25°C±0.1°C and pH=5.55.

	10^3 [RNH_2]$_F$	10^2 k_{obs} / s^{-1}
1	3.00	0.40
2	7.50	1.05
3	12.0	2.01
4	16.5	2.42
5	21.0	2.96
6	25.5	3.30
7	30.0	4.39

Figure S1. Plot of k_{obs} against free amine concentration $[RNH_2]_F$ for the reaction of pyrimidine with hydrazine at three pH values in aqueous solution at 25.0 °C and ionic strength 0.2M (KCl). R-square = 0,986; standard error = 0,086 and N = 20.

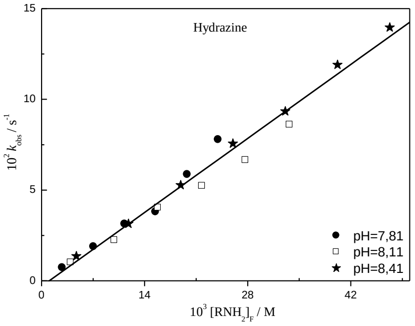

•

Figure S2. Plot of k_{obs} against free amine concentration $[RNH_2]_F$ for the reaction of pyrimidine with N-methyl hydroxylamine at three pH values in aqueous solution at 25.0 °C and ionic strength 0.2M (KCl). R-square = 0,976; standard error = 0,120 and N = 21.

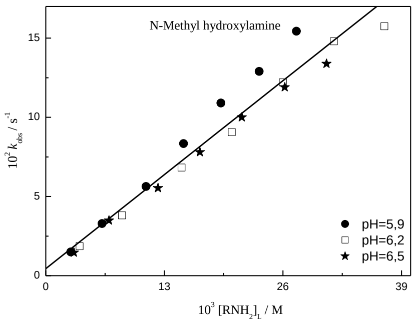

Figure S3. Plot of k_{obs} against free amine concentration $[RNH_2]_F$ for the reaction of pyrimidine with hydroxylamine at three pH values in aqueous solution at 25.0 °C and ionic strength 0.2M (KCl). R-square = 0,950; standard error = $5,720 \times 10^{-5}$ and N = 20.

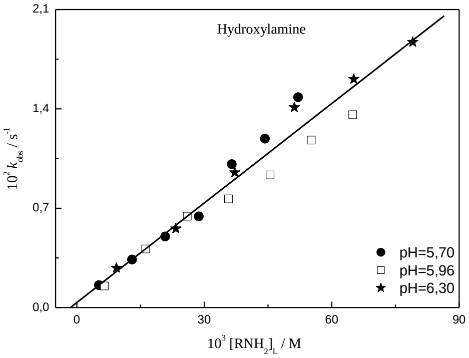

Figure S4. Plot of k_{obs} against free amine concentration $[RNH_2]_F$ for the reaction of pyrimidine with N,N-dimethyl hydroxylamine at three pH values in aqueous solution at 25.0 °C and ionic strength 0.2M (KCl). R-square = 0,969; standard error = 0,086 and N = 19.

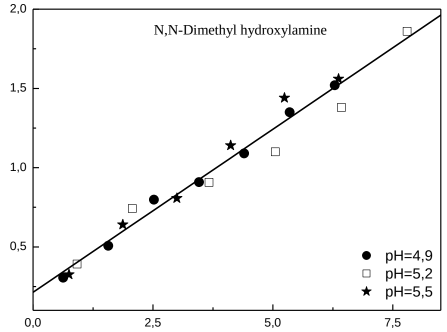

Figure S5. Plot of k_{obs} against free amine concentration $[RNH_2]_F$ for the reaction of pyrimidine with N,O-dimethyl hydroxylamine at three pH values in aqueous solution at 25.0 °C and ionic strength 0.2M (KCl). R-square = 0,985; standard error = 0,012 and N = 21.

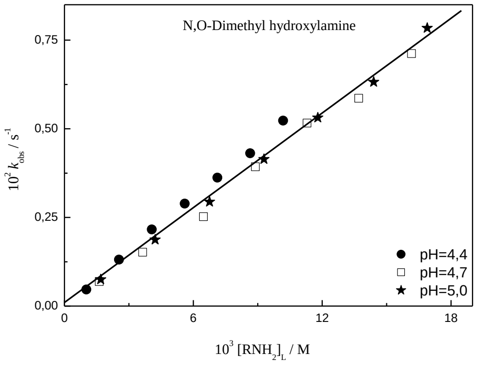

Figure S6. Plot of k_{obs} against free amine concentration $[RNH_2]_F$ for the reaction of pyrimidine with methoxylamine at three pH values in aqueous solution at 25.0 °C and ionic strength 0.2M (KCl). R-square = 0,968; standard error = 8,213x10^{-4} and N = 20.

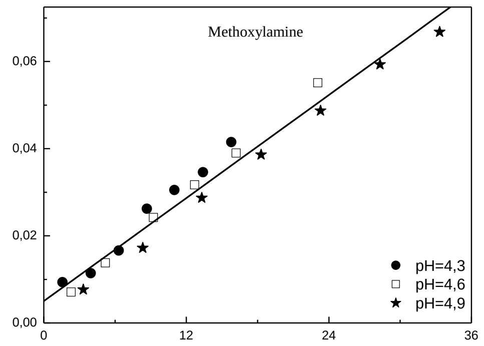

Figure S7. Plot of k_{obs} against free amine concentration $[RNH_2]_F$ for the reaction of pyrimidine with 4-phenylenediamine at three pH values in aqueous solution at 25.0 °C and ionic strength 0.2M (KCl). R-square = 0,977; standard error = 0,108 and N = 21.

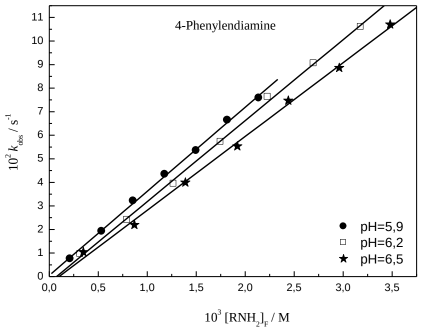

Figure S8. Plot of k_{obs} against free amine concentration $[RNH_2]_F$ for the reaction of pyrimidine with 4-methoxyaniline at three pH values in aqueous solution at 25.0 °C and ionic strength 0.2M (KCl). R-square = 0,971; standard error = 0,026 and N = 20.

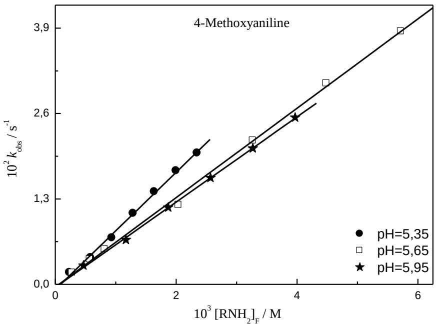

Figure S9. Plot of k_{obs} against free amine concentration $[RNH_2]_F$ for the reaction of pyrimidine with 4-methylaniline at three pH values in aqueous solution at 25.0 °C and ionic strength 0.2M (KCl). R-square = 0,988; standard error = 0,006 and N = 21.

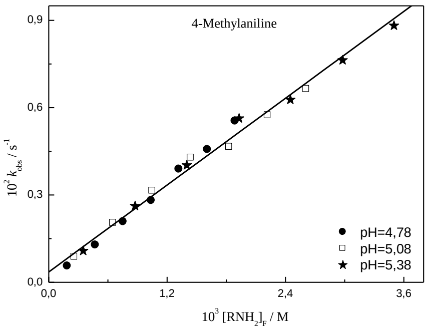

Figure S10. Plot of k_{obs} against free amine concentration $[RNH_2]_F$ for the reaction of pyrimidine with aniline at three pH values in aqueous solution at 25.0 °C and ionic strength 0.2M (KCl). R-square = 0,996; standard error = 0,001 and N = 21.

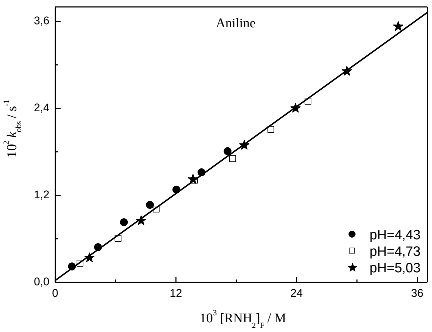

Figure S11. Plot of k_{obs} against free amine concentration $[RNH_2]_F$ for the reaction of pyrimidine with 3-methoxyaniline at three pH values in aqueous solution at 25.0 °C and ionic strength 0.2M (KCl). R-square = 0,926; standard error = 0,003 and N = 21.

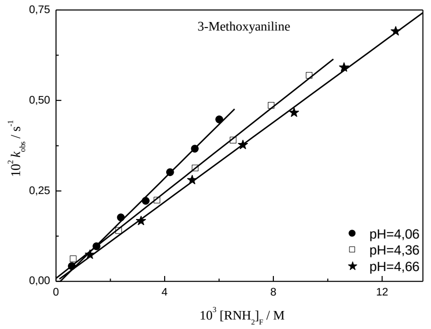

Figure S12. Plot of k_{obs} against free amine concentration $[RNH_2]_F$ for the reaction of pyrimidine with 3-aminoacetophenone at three pH values in aqueous solution at 25.0 °C and ionic strength 0.2M (KCl). R-square = 0,977; standard error = 9,540x10^{-4} and N = 19.

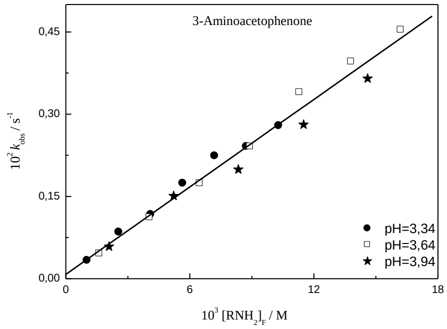

Figure S13. Plot of k_{obs} against free amine concentration $[RNH_2]_F$ for the reaction of pyrimidine with phenylhydrazyne at three pH values in aqueous solution at 25.0 °C and ionic strength 0.2M (KCl). R-square = 0,966; standard error = 0,006 and N = 19.

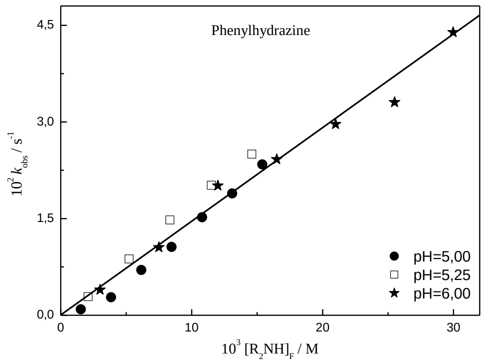

Supplement: Supplementary file 1 [file DataSheet1.pdf]
